# Potential Contribution of Type I Alveolar Epithelial Cells to Chronic Neonatal Lung Disease

**DOI:** 10.3389/fped.2014.00045

**Published:** 2014-05-19

**Authors:** Henry J. Rozycki

**Affiliations:** ^1^Division of Neonatal Medicine, Children’s Hospital of Richmond at Virginia Commonwealth University, Richmond, VA, USA

**Keywords:** alveoli, bronchopulmonary dysplasia, RAGE receptor, inflammation, innate immunity

## Abstract

The alveolar surface is covered by large flat Type I cells (alveolar epithelial cells 1, AEC1). The normal physiological function of AEC1s involves gas exchange, based on their location in approximation to the capillary endothelium and their thinness, and in ion and water flux, as shown by the presence of solute active transport proteins, water channels, and impermeable tight junctions between cells. With the recent ability to produce relatively pure cultures of AEC1 cells, new functions have been described. These may be relevant to lung injury, repair, and the abnormal development that characterizes bronchopulmonary dysplasia (BPD). To hypothesize a potential role for AEC1 in the development of lung injury and abnormal repair/development in premature lungs, evidence is presented for their presence in the developing lung, how their source may not be the Type II cell (AEC2) as has been assumed for 40 years, and how the cell can be damaged by same type of stressors as those which lead to BPD. Recent work shows that the cells are part of the innate immune response, capable of producing pro-inflammatory mediators, which could contribute to the increase in inflammation seen in early BPD. One of the receptors found exclusively on AEC1 cells in the lung, called RAGE, may also have a role in increased inflammation and alveolar simplification. While the current evidence for AEC1 involvement in BPD is circumstantial and limited at present, the accumulating data supports several hypotheses and questions regarding potential differences in the behavior of AEC1 cells from newborn and premature lung compared with the adult lung.

## Introduction

Bronchopulmonary dysplasia (BPD) is a common and serious complication that affects up to 25% of infants born at <1500 g. In fact, prematurity is the strongest risk factor for BPD (1). When the premature lung is exposed to damage and stressors, from high oxygen levels and/or mechanical ventilation and its associated volutrauma, it can trigger a strong inflammatory response (2). When this occurs during a vulnerable period of lung development, prior to the alveolar stage (before 32–34 weeks gestation in humans), there is an arrest of development leading to a decrease in secondary septal formation and alveolarization. As a result, the lungs of survivors of prematurity with BPD have relatively simplified lungs, with decreased alveoli and surface area (3).

The story of research into the pathogenesis of BPD has followed the larger story of biomedical research, moving from pathology and physiology, to clinical risks and therapies and now an increasing focus on the cellular and molecular reasons why the premature lung is both so susceptible to damage, and why it reacts in the way it does. For example, as outlined in a recent review (3), one of the clinical risk factors, hyperoxia, promotes apoptosis, affects the growth factors and other signals that are the developmental cross-talk between mesenchyme and epithelium that regulate alveolarization (4).

There are over a dozen cell types in the lung parenchyma, including fibroblasts, endothelial cells, dendritic cells, alveolar macrophages, airway epithelial cells, Type II (AEC2), and Type I epithelial cells (AEC1). Numerous studies have focused on each of these cell types in relation to BPD. Of these, the AEC1 has perhaps been the least examined, primarily because the cell was presumed to be terminally differentiated, unable to proliferate and limited in its potential responses to events like premature birth and injury. This was reinforced by the difficulty of isolating and working with AEC1 cells. As a result, there is limited understanding about the cell in relation to any significant role it may play in BPD. The purpose of this review is to bring together work done over the last 15 years that has deepened and expanded our appreciation of the AEC1 cell, and how these findings point to a potential role in the development of BPD. These roles are hypothesized and questions generated to begin to address these hypotheses.

## The AEC1 Cell – Gas Exchange and Ion/Fluid Flux

**Figure 1:**
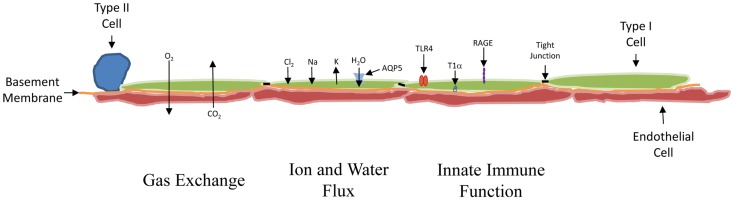
**Schematic of the distal lung**. From left to right: the cuboidal AEC2 cell was the presumed source of new AEC1 cells. In at least 50% of the lung, the AEC1 cell (green) shares a basement membrane (orange) with the capillary endothelial cell (red), permitting the exchange of O_2_ and CO_2_ to occur across them. The AEC1 cell is also involved in the movement of water and ions. The ions move via a variety of channels. The tight junctions (black bar) inhibit water movement between cells. Water does move into the AEC1 cell from the alveolar (upper) side through AQP5 channels. The AEC1 cells express receptors capable of promoting an inflammatory response, including TLR4 and RAGE. The T1α receptor, which localizes to the basal side of the AEC1 cell, is probably used for anchoring the cell. It is specific for the AEC1 cell and often used to develop pure preparations of AEC1 cells for study.

Morphologically, the AEC1s are large, thin, and flat (Figure 1). While they make up just over 10% of the cells within an alveolar unit in the normal adult human lung, compared with 18% for the AEC2 cell, they cover 16 times the surface area (5). They are situated in the lung in proximity to the capillary endothelium, separated by either a shared basement membrane or a layer of extracellular matrix. In approximately half of the lung surface area, there is a very thin shared basement membrane structure, and this is felt to be the most important site for gas exchange (6).

The cells also function to help maintain ion and fluid balance and control their flux at the air–liquid interface (7). Proper lung function involves the alveolar lining fluid, which creates the air–liquid interface. Evidence supports the critical role of the AECI cell in the maintenance of the optimal amount of this fluid. First, the tight junctions between the cells limit the overall movement of ions and water (8). These junctions can be disrupted by stretch, which could explain how barotrauma might lead to edema (9). Second, the cell contains a number of ion transport channels, including the amiloride-sensitive epithelial sodium channels (ENaC), CFTR chloride channels, and a variety of potassium channels, which help maintain the electrochemical gradient needed for ion movement (10). Finally, one of the proteins used to characterize the Type I cell is aquaporin-5 (AQP5). Depending on the species studied, it is present in late gestation or early post natal life almost exclusively in the AEC1 cell (11), and it is responsible for most of the water flow across the lung epithelium (12). AQP5 mRNA levels rise acutely in newborn mice exposed to hyperoxia and can remain elevated if the rodents are returned to room air (13). This might help explain the increase in lung water and decreased compliance seen in hyperoxic premature lung injury (14).

## Are AEC1 Cells Present in the Premature Lung?

The purpose of this paper is to explore whether the AEC1 cell contributes to BPD. As a first step, one must first show that AEC1 cells are present at the appropriate time. In rodents, the large flat AEC1 begins to appear in the late canalicular period and increases in number as the internal surface area increases during the saccular and, especially, in the alveolar stage of mammalian lung development. Until recently and based on morphology, it has generally been felt that the cell appears at around the same time as the AEC2 alveolar epithelial cell, at least in mice and rats. There is some evidence that this is under the control of the Wnt/b-catenin system since absence of b-catenin in lung epithelium inhibits lung branching and respiratory epithelium differentiation and overexpression causes epithelial hyperplasia (15). *In vivo* studies also support roles for the transcription factors Fox2a and TTF-1 and C/EBPa in terminal differentiation of respiratory epithelium. Recent reports show that Hippo kinases upstream of Fox2a are also involved (16).

In contrast to rodents, studies in larger mammals such as sheep, using electron microscopy to identify the AEC1 cells, indicate that the cells are present far earlier in development (17). At 91 days (term = 145–147 days), 4.8% of the cells appeared to be AEC1 cells. This was higher than the 1.2% of cells that were classified as AEC2 cells. Furthermore, at 111 days, the proportion of AEC1 to AEC2 cells was 63 and 4.3%, respectively.

It was only in the late 1990s that AEC1-specific cell proteins were identified, and antibodies developed for these markers. These markers were reviewed in 2003 (18). Two of the most prominent are T1α, which is expressed in other tissues such as the kidney and the brain but which, in the lung, is exclusively found in AEC1 cells, and RAGE, the receptor for advanced glycation end-products, which is also found in many organs but only on AEC1 cells in the lung.

T1α knockout mice that die at birth of respiratory failure, have far fewer AEC1 cells, increased cell proliferation, and decreased alveoli, indicating a role for T1α and for AEC1 in overall lung development (19). T1α protein in whole lung homogenates is low in rat lungs until just prior to delivery, which correlates to the morphological appearance referred to above. However, by immunohistochemistry, lung staining is visible in the tubules that are just proximal to the developing distal end of the branches by embryonic day 17, and only extends to the distal buds by embryonic day 20 (20). Whether the T1α positive cells seen before the active increase in both T1α protein and AEC1 cell number are the source of the AEC1 differentiation is not known. Information regarding RAGE is outlined below.

## Where Do AEC1 Cells Come from?

Based on several lines of evidence, it has generally been held that the AEC2 cell is the source of the AEC1 cell. First, in *in vivo* injury models dating back to the early 1970s, there was a loss of AEC1 cells, followed by a replenishment, but only AEC2 cells showed signs of proliferation. Evans et al. demonstrated, more than 40 years ago, that when lungs were exposed to NO_2_ and the lung epithelium injured, it was the AEC2 cell that took up tritiated thymidine, indicating cell division, while the remaining AEC1s were quiescent and that the sister cells derived from the dividing AEC2 cell often became new AEC1 cells (21). Second, when cultured on plastic, AEC2 cells become flattened and begin to express proteins that are present in AEC1 cells but not in AEC2 cells, including T1α (22), AQP5 (23), and caveolin-1 (24). This transdifferentiation is directed, in part, by switching from BMP-predominant to TGF-β predominant signaling (25). Studies of lineage-labeling AEC2 cells demonstrated that under conditions of injury, AEC1 cells came from AEC2 parents (26). The inability to demonstrate AEC1 cell proliferation led to the conclusion that these cells were terminally differentiated and had lost their ability to divide, so they must come from another source.

There are several observations and experiments, however, which call this conclusion into question. As noted above, in electron microscopy studies in sheep fetuses, AEC1 cells are present in larger proportion than AEC2 cells very early in gestation. In another study in sheep, the same group of investigators showed that expansion of lung volumes by tracheal occlusion in the fetus increased the proportion of AEC1 cells from 63 to almost 90% but that if the lung was then deflated, the proportion of AEC1 cells fell, while AEC2 cells increased. This was accompanied by the increased appearance of what the authors termed an “intermediate cell.” Along with hypothesizing that AEC1 cells have the capacity to transdifferentiate to AEC2 cells, the authors proposed that this intermediate cell, and not just AEC2 cells, may be a potential source of both AECI and AEC2 cells (27).

The concept that there are lung cells that have the capacity to become either AEC1 or AEC2 cells is strongly supported by work summarized this year in a paper in *Nature* from Desai et al. (28) in which they describe how, in mice, markers of AEC1 cells appear 5 days before saccularization; how the expression pattern of cell-specific markers (including T1α and RAGE for AEC1 and surfactant protein C, Nkx2.1, and ABCA3 for AEC2 cells) is consistent with a precursor cell that expresses some of each class of markers before further differentiating; how the clonal expansion of distal airway cells labeled at embryonic day 15.5 with green fluorescent protein (GFP) demonstrated the label in both cell types in newborn and older mice; that no one has described the intermediate cell between the AEC2 and the AEC1 in which one should be able to see both the flattened morphology of the AEC1 cell type along with residual lamellar bodies that mark the AEC2 cell (this, in fact, is described in the fetal sheep (27)); how, when AEC2 cells were labeled with GFP, the label did not appear in AEC1 cells. This argues strongly that the normal source of AEC1 cells is not the AEC2 cell but rather what they term a bipotent progenitor cell. This progenitor cell itself may be an intermediate step, derived from what looks morphologically like an alveolar epithelial stem cell (17).

These authors also used AEC2 cell lineage-labeling to look at an injury model and found that after hyperoxia in 2-month-old mice, the number of labeled AEC1 cells went up significantly. This may be why even recent reviews have maintained that AEC1 cells are derived from AEC2 cells, i.e., the original studies involved injury models (21).

## Damage to AEC1 Cells

BPD is precipitated by damage done by premature birth, volutrauma, and oxygen toxicity. How do AEC1 cells react to injury?

Because they cover so much of the surface of the lung, AEC1cells are at risk for environmental injury. Bleomycin is a common method to induce injury, both acute and chronic, in animal models. Exposure initially denudes the lung of AEC1 cells and leads to an overall loss of these cells in the subsequent fibrotic lung (26).

Regarding hyperoxic injury, in 1968, Bowden et al. showed that the AEC2 cell was relatively spared from hyperoxic injury compared to AEC1 (29). In another study, using adult rats, 7–14 days exposure to 85% oxygen did not affect the number of AEC1 cells, whereas the number of AEC2 cells was markedly increased. However, by electron microscopy, the intracellular organelles such as endoplasmic reticulum, Golgi apparatus, and mitochondria within the AEC1 population were all hypertrophied, as if the cells were geared up to produce in response to the high oxygen concentration (30). It may be that AEC1 susceptibility to oxygen-induced injury is species specific. When adult baboons were exposed to 100% oxygen, injury was first seen in endothelial cells followed by AEC1 cells, which demonstrated a necrosis pattern (31). This is similar to the *in vitro* effects of high oxygen on the lung adenocarcinoma cell line A549 (32). In contrast, in newborn mice, exposure to 100% O_2_ increased the mRNA for T1α and AQP5, which are AEC1-specific, implying that AEC1 cells did not die, but either increased in number or turned on the expression of these genes (13). Overall, these models demonstrate that AEC1 cells have the capacity to respond to oxygen stress, but the type of response has not been consistently observed.

Mechanical ventilation, primarily consisting of cyclic stretching from volume, is a second primary clinical association with BPD. The direct relationship between stretch and AEC1 cells has not been well-studied, probably because of the difficulty in isolating and growing AEC1 cells *in vitro*. There is, however, some indirect evidence for AEC1 responsiveness to stretch.

Cyclic stretching of lung epithelial cells allowed to differentiate to Type I phenotype *in vitro* caused an increase in their permeability, and this was blocked by the addition of either an antioxidant or an NF-κB activation inhibitor (33). Cyclic stretch of the lung epithelial cell line A549, which has both AEC2 and AEC1 properties [for example, it expresses RAGE (34)] provoked increases in pro-inflammatory cytokine production (35). In another study of A549 cells, investigators demonstrated an increase in HMGB1 production and release (36). HMGB1 is a damage-associated molecule released by stressed and necrotic cells and can stimulate inflammation (37).

The susceptibility of AEC1 cells to changes induced by stretch may also be dependent on the (a) type of stretch or expansion and (b) on the degree of prematurity. This is illustrated by the studies of continuous static expansion in sheep. When done in the fetus, the proportion of AEC1 cells went up markedly (27), but this was not seen with high versus low PEEP ventilation in 2-week-old lambs (38).

The evidence for AEC1 response to hyperoxia is stronger than for volutrauma, which awaits more direct experimentation on AEC1 cells, but taken together, it is very likely that AEC1 cells do respond to the type of stimuli most closely associated with BPD.

## Innate Immune Function

**Figure 2:**
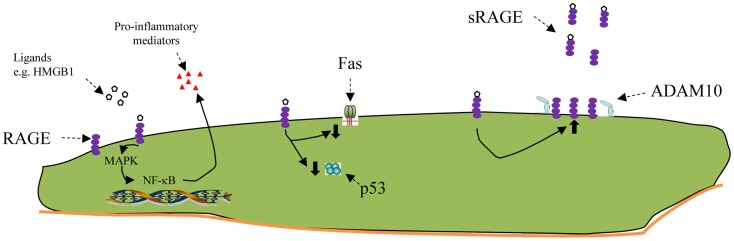
**Schematic of some RAGE functions**. From left to right: engagement of the RAGE receptor with ligands such as HMGB1 activates intracellular pathways, including several MAPkinases, resulting ultimately in activation of NF-κB. This, in turn, can promote the expression and export of pro-inflammatory mediators. RAGE activation has also been shown to inhibit apoptosis, both by decreasing FAS (CD95) on the cell surface and by inhibiting p53. RAGE activation increases the production of more RAGE. Soluble Rage (sRAGE) is a truncated variant of whole RAGE, missing the transmembrane portion, which is snipped off by a cell surface metallopeptidase, ADAM10. Once released, sRAGE can bind to free RAGE ligands and make them unavailable to bind to membrane-bound RAGE. In newborn animals exposed to LPS *in utero*, or hyperoxia after birth the amount of soluble RAGE is diminished, which would lead to less attachment of the sRAGE to the pro-inflammatory ligands, making them more available to attach to pro-inflammatory receptors like mRAGE at the left.

As noted, the earliest stages in the development of BPD are associated with inflammation (2) (Figure 2). Until AEC1 cells could be studied independently, their immune and inflammatory functions were unknown. In 1988, Dobbs et al. described a protein, which they called rTI40 that was specific for AEC1 cells (22). In 1995, Rishi et al. cloned the T1α gene and showed how, in the lung, it was exclusively expressed in AEC1 cells (39), and it turned out that rTI40 and T1α are the same. This led to the use of specific antibodies to prepare highly enriched samples of AEC1 cells from rats and mice. Freshly isolated AEC1 cells, it turned out, were different from AEC1 cells derived from *in vitro* differentiation of AEC2 cells. They differ in their molecular phenotype (40). They have the capacity to proliferate (41). A recent review provides details of the many features that culture of AEC1 has revealed (42).

Among the newly revealed capacities of the AEC1s is an innate immune effect. This is not surprising given that these cells have, after the skin, the largest exposure to the environment. The cells express toll-like receptor 4, which reacts to bacterial products and produce cytokines such as TNF-α, IL-β, and IL-6 after exposure to LPS both *in vivo* and *in vitro* (43). In fact, some authors believe that the AEC1 cell is a more important source of pro-inflammatory signaling than the AEC2 cell, which has traditionally been thought of as the distal lung cell responder to noxious environmental stimuli (44).

## The RAGE Receptor

One potentially intriguing characteristic of the AEC1 is its expression of the RAGE, a member of the immunoglobulin superfamily (Figure 2). RAGE is expressed on the surface of many cell types, but the highest levels are found in the lung, specifically on the AEC1 (45). In rodents, limited amounts of RAGE are present before E18.5, at which point the levels rise steadily, especially after P4 when the animal enters the alveolar phase (46). A note of caution must be added when looking at studies that compare RAGE or any other AEC1 marker, between newborn and older animals, especially rodents. These measures are usually normalized to total protein or, in the case of mRNA, to a housekeeping gene, and are derived from whole lung samples. Since the number of AEC1 cells only begin to rise exponentially at the alveolar phase, the total amount of the receptor might be low, but the relative amount of the receptor to the cells capable of expressing it could be completely different.

Investigators have described several aspects of the biology of RAGE that potentially implicate it in the development of BPD in newborn infants.

Aside from the glycation end-products which gave the receptor its name, ligands for RAGE include damage-associate products, including HMGB1 (47), which have been associated with BPD (48) and, as noted above, is released from damaged lungs and A549 cells (36).

Upon stimulation, RAGE signals are transduced through MAPK and NF-κB pathways and promote a variety of cellular functions. Relevant to newborn lung disease, these functions include the production of pro-inflammatory cytokines, as well as apoptosis (49). Apoptosis is an important mechanism for hyperoxic lung epithelial injury (50). Reynolds et al. demonstrated that adult RAGE-null mice survived longer and had diminished inflammation and inflammatory signals after 4 days in 75% F_i_O_2_ (51), implicating the RAGE receptor in pathways leading to hyperoxic pulmonary injury.

After the initial inflammatory stage in BPD, there is inhibition of normal development, with decreased secondary septal formation and decreased alveolar development. In both adult and late fetal rats, overexpression of RAGE leads to simplification of lung architecture, leading to a picture which resembles BPD (49, 52). Conditional overexpression of RAGE in surfactant protein C expressing cells late in gestation decreased the number of AEC1 cells and seemed to make their attachment to the basement membrane far less secure (53).

It would, therefore be tempting to invoke a role for RAGE in the pathophysiology of BPD, first in the inflammatory phase and then in the developmental arrest/alveolar inhibition phase. It must be noted, however, that some data raise questions about this hypothesis.

Rats born to mothers who had received intramaniotic LPS (to create chorioamnionitis) and who were then reared in hyperoxia had more collagen and TGF-β but decreased RAGE mRNA and sRAGE protein than controls (54). This is one among the number of animal models of BPD and may not be the best, as the role of chorioamnionitis in BPD has become controversial (55). On the other hand, when newborn rats were exposed to 95% oxygen for 2 days, there was no effect on either membrane-bound or sRAGE. Only after 8 days of hyperoxia was there a decrease in the soluble form only, again for whole lung rather than relative to AEC cell number (56).

One of the interesting and unique aspects of RAGE behavior, and why it may be important to take into account what measurements are being normalized to, is that rather than downregulating expression, as often happens to receptors, engagement with its ligands leads to increases in RAGE mRNA and protein levels. That this does not lead to uncontrolled inflammation may be because RAGE exists primarily in two forms, a transmembrane receptor and a shorter soluble form, which is released locally and soaks up ligands, making them unavailable for binding to the cell-associated RAGE receptor. One study has examined lung sRAGE levels and found them lower on the first day of life in premature infants born to mothers with histological chorioamnionitis. The authors speculated that its absence contributed to a pro- versus counter-inflammatory imbalance that has been associated with BPD (57). Thus, too much RAGE might interfere with lung development, while too little, at least of the soluble form, might enhance the inflammatory response.

## Hypothetical Roles for AEC1 Cells in BPD

Based on what has been outlined here, there are several ways in which AEC1 cells might play a role in the development of BPD. Perhaps most importantly, the cells are present and developing (rapidly at the alveolar stage), so that perturbations could have significant implications. It must be stated up front, however, that the hypothesis that AEC1 cells are more sensitive to damage and that they are preferentially lost, *without* having any secondary effects, is certainly plausible, given the current state of the evidence.

- The absence of AEC1 cells during development causes severe disruption of lung development distally, at the terminal air sacs (52). This means that their presence may be critical. Alveolar development is controlled by signals that go back and forth between the mesenchyme and the invading/growing epithelium as well as between epithelial cell types (4). Since these cells are among the most susceptible to injury, one can hypothesize that damage to the AEC1 cells and/or their progenitors could interfere with the signaling cross-talk, becoming one contributing factor to the disruption of pulmonary development in BPD.
- The early pro-inflammatory state of the premature lung that eventually can be diagnosed as BPD is one area in which AEC1 cells might be acting. The cells have the capacity to produce several of the mediators that have been associated with BPD, including TNF-α, IL-β, and IL-6 (43). They can produce HMGB1, a ligand for RAGE. They, in fact, are the main, if not sole, cell type expressing RAGE. To date, however, there is no evidence that inflammatory mediators in either human neonates or in animal models of BPD come from AEC1s, but until recently it was not possible to study AEC1 cells alone. There are as yet no studies yet examining how the innate immune response in AEC1s might change over development, by comparing cells from the pre-alveolar stage to ones in the alveolar stage and from adult lungs.
- The potential role of the RAGE receptor is also intriguing. Not only can its activation promote inflammation but also too much RAGE (which is meant to mimic too much RAGE activation) can lead to the kind of simplified lung morphology seen in established BPD (49, 52). This is what was seen in the studies where RAGE expression was begun 2–3 days before delivery in mice (49), coinciding with the saccular stage of lung development. Infants who are exposed to oxygen and volutrauma at this stage are at the highest risk for BPD.

There is, as yet, no evidence for any role for RAGE in BPD, except indirectly, as one of its ligands, HMGB1, is elevated in the lungs of babies who get BPD. HMGB1 can also bind to the toll-like receptors 2 and 4 and trigger inflammatory mediator expression so this observation is not conclusive.

On the other side of the coin, a decrease in RAGE, especially the soluble form, might be a way by which RAGE contributes to lung inflammation. Decreased sRAGE can lead to increased inflammation as stimuli become more available to bind to pro-inflammatory receptors on alveolar macrophages, AEC2 cells, and AEC1s. The observations about the rat chorioamnionitis/hyperoxia model (54), and the prolonged newborn hyperoxia model (56), both of which had decreased sRAGE, are consistent with this hypothesis. Alternatively, this might again demonstrate an increased overall loss of AEC1 cells and not have any direct effects.

## Some Questions Raised

Using current techniques, up to 99% pure AEC1 cells can be isolated from rat lungs (42). Applying and modifying these methods to the newborn and even preterm animal can provide the means to begin answering some of the questions raised by the hypotheses in the previous section. Some of the early questions that might be explored include:

- Are newborn (pre-alveolar) rodent AEC1 cells more sensitive to damage from hyperoxia?
- Can AEC1 cells raised in culture react to cyclic stretch and does this response differ in cells from younger animals?
- Does the absence of AEC1 cells or of their progenitor cell cause a similar pathology to BPD? Tying a conditional lethal gene to RAGE or T1α expression could address this.
- Do younger cells have an increased response to pro-inflammatory stimuli such as LPS or HMGB1 *in vitro*?
- Is there a difference between RAGE density on younger and older AEC1 cells? Is there a difference in the RAGE response to stimulation by age? Is there a difference in the release of sRAGE?

There are, of course, other questions that can be asked leading to other investigations. With the availability of established techniques for acquiring AEC1 cells and the tantalizing but very incomplete data reviewed here, this is a good time to put effort into studying AEC1 cells and determining what contribution they make to the development of BPD.

## Conflict of Interest Statement

The author declares that the research was conducted in the absence of any commercial or financial relationships that could be construed as a potential conflict of interest.
